# Bilateral Unfused Medial Process of Calcaneal Apophysis associated with Lower Extremity Malalignment: A Case Report

**DOI:** 10.2174/0115734056386404250730094252

**Published:** 2025-08-04

**Authors:** Yu Sung Yoon

**Affiliations:** 1 Department of Radiology, School of Medicine, Kyungpook National University, Kyungpook National University Hospital, Daegu, South Korea

**Keywords:** Calcaneus, Apophyseal development, Anatomical variation, Lower extremity deformities, Developmental bone diseases, Genu valgum

## Abstract

**Introduction:**

The calcaneal apophysis develops through a complex ossification process during childhood growth, with multiple secondary ossification centers emerging in distinct temporal and spatial patterns. Its ossification patterns, fusion process, and associated pediatric injuries and osteochondral conditions have been well documented in the literature. This report presents a previously unreported case of bilateral unfused medial process of calcaneal apophysis incidentally discovered in an adolescent patient during evaluation for genu valgum. We aim to describe this unique presentation and discuss potential pathogenic mechanisms underlying this distinctive anatomical variation.

**Case Presentation:**

A 12-year-old female patient was referred for idiopathic bilateral genu valgum and ankle valgus deformity management, with no prior treatment history or symptoms. Initial radiographs showed bilateral symmetric deformities, while CT revealed bilateral separated apophyses (Lt.; 8.8 mm, Rt.; 9.4 mm) at the medial process of the calcaneus with sclerotic margins. No underlying bone pathology or structural abnormalities were identified.

**Discussion:**

The bilateral unfused medial processes of the calcaneal apophysis in this patient represent a novel anatomical variation occurring alongside coxa valga and genu valgum. Biomechanical research indicates that hindfoot eversion increases medial heel pressure by 15%, with valgus alignment generating 11-12% higher medial heel pressure compared to lateral regions. These altered pressure patterns may influence apophyseal development. Normally, the medial process develops around age 9-10 and fuses 12-24 months later, with complete fusion by ages 14-16 in females. Our patient's bilateral persistence of unfused apophysis deviated significantly from this timeline. This selective non-fusion pattern differed from known pathological conditions, thus warranting further investigation through systematic studies.

**Conclusion:**

This case highlights a rare anatomical variant of bilateral unfused medial calcaneal apophyses discovered incidentally in an adolescent. While the clinical significance remains uncertain, the bilateral and symmetric nature of these findings suggests a developmental variant rather than a pathological condition. This observation contributes to our understanding of variations in calcaneal apophyseal development.

## 1 INTRODUCTION

The calcaneal apophysis, a secondary ossification center located on the posterior aspect of the calcaneus, undergoes a complex developmental process during childhood growth. Previous studies have established the typical timeline of ossification, occurring between ages 5-7 in females and 7-8 in males, with complete fusion observed between ages 14-16 in females and 16-20 in males [1, 2].

Current literature describes the biomechanical significance of the calcaneal apophysis [3-5]. Research has demonstrated that the ossification centers are initially situated in the lower third of the calcaneus, with their number varying from a single large center to multiple smaller ones. [2, 6]. These ossification centers create critical attachment sites for the abductor hallucis, flexor digitorum brevis, and plantar aponeurosis medially, as anatomical studies have noted the importance of these soft tissue attachments for foot biomechanics [3, 7].

Despite extensive radiographic and MRI studies documenting normal ossification patterns of the calcaneal apophysis [1, 2], the literature lacks comprehensive documentation of anatomical variants, particularly regarding unfused medial processes. This knowledge gap becomes especially relevant in the context of lower extremity malalignment conditions, such as coxa valga and genu valgum, where altered biomechanical forces may potentially influence calcaneal apophyseal development patterns [3, 8, 9].

This report describes an incidental finding of a bilateral unfused medial process of the calcaneal apophysis in a patient with idiopathic coxa valga and genu valgum. The case presents an opportunity to expand the current understanding of anatomical variants in calcaneal apophyseal development and their potential associations with lower extremity alignment abnormalities. Such observations may provide valuable insights into both developmental anatomy and clinical practice considerations.

## 2 CASE PRESENTATION

A 12-year-old female patient was referred to our institution for surgical management of idiopathic genu valgum and ankle valgus deformity. The patient presented with bilateral lower extremity deformities but reported no specific osteoarticular symptoms in the knees, ankles, or feet. There was no previous history of surgical intervention, orthosis application, or medical treatment. Physical examination revealed bilateral genu valgum and ankle valgus deformity without any evidence of joint instability, tenderness, or limited range of motion.

**Fig. (1):**
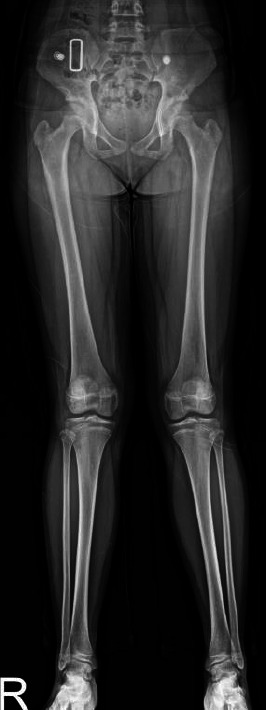
Whole extremity AP radiograph of a 12-year-old female. Whole extremity AP radiograph shows bilateral genu valgum and ankle valgus deformity.

**Fig. (2):**
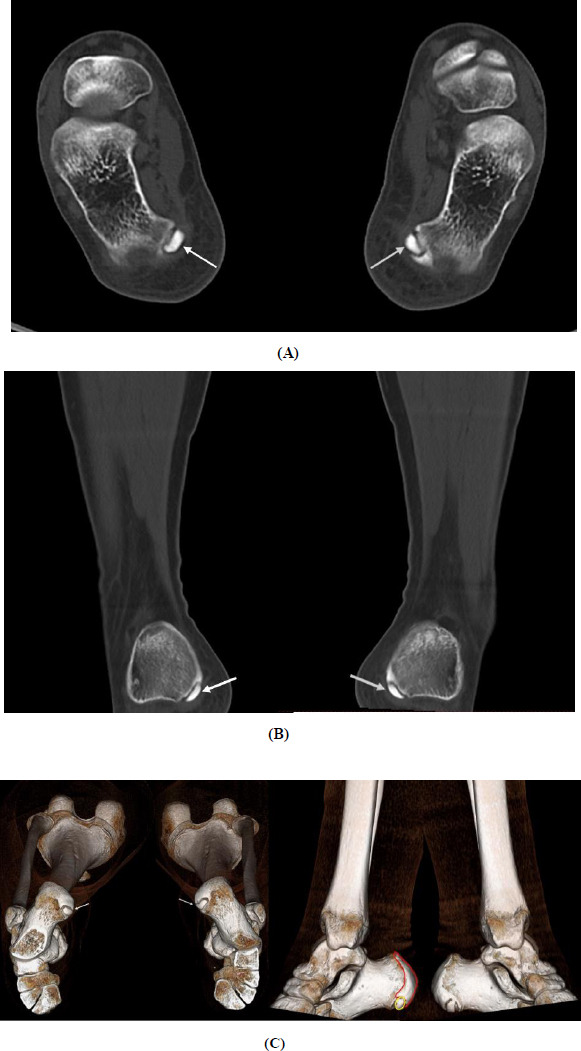
3-dimensional non-contrast computed tomography (CT) scan of a 12-year-old female. Both axial (**A**), coronal (**B**) reformatted images, and VR images (**C**) show bilateral unfused medial calcaneal apophyses (white arrows and yellow circle) with well-defined corticated margins. The ossicles measured 8.8 mm and 9.4 mm in maximal diameter on the left and right sides, respectively. (Note. Red circle: fused calcaneal apophysis)

Initial full-length standing anteroposterior (AP) radiographs demonstrated bilateral genu valgum and ankle valgus deformity (Fig. **1**). Subsequent CT examination of the ankles revealed bilateral unfused medial calcaneal apophyses (Fig. **2**). The unfused ossicles demonstrated well-corticated margins and measured 8.8 mm and 9.4 mm in maximum diameter on the left and right sides, respectively. These ossicles maintained consistent density with the adjacent calcaneal body, without evidence of fragmentation or irregularity. Three-dimensional reconstruction confirmed their anatomical location at the medial process of the calcaneus bilaterally. No inflammatory changes, bone erosion, or abnormal enhancement were observed in the surrounding soft tissue or adjacent bones.

**Fig. (3):**
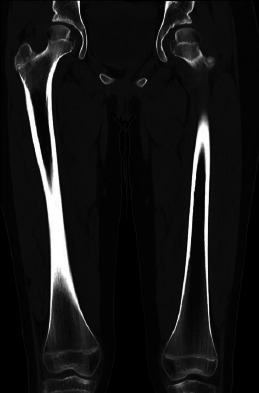
Coronal reformatted CT images of both femurs in a 12-year-old female.CT imaging of the femurs demonstrates bilateral genu valgum. No evidence of physeal abnormality, bone dysplasia, or other structural abnormalities was identified.

CT examination of the femur was performed to evaluate the extent of the valgus deformity and to rule out any underlying bone pathology (Fig. **3**). The scan confirmed bilateral genu valgum without evidence of physeal abnormality, bone dysplasia, or other structural abnormalities.

Based on these clinical and radiological findings, the patient underwent bilateral surgical correction. The procedure consisted of medial hemiepiphysiodesis of the distal femur using 8-figure hinge plates with screw fixation at the medial epimetaphysis. Concurrent bilateral medial malleolar hemiepiphysiodesis was performed using transphyseal screw fixation. The surgical approach was selected to achieve gradual correction of both the femoral and ankle deformities through guided growth.

## 3 DISCUSSION

The bilateral, unfused medial processes of the calcaneal apophysis observed in this patient represent a previously unreported anatomical variation, which occurs concurrently with coxa valga and genu valgum. This alignment results in medial displacement of the mechanical axis, raising important questions about biomechanical influences on apophyseal development. Given the distinctive anatomical characteristics and bilateral occurrence of this ossification center, we propose the term *'Os calcanei apomedialis*' to describe this unique anatomical variant. This nomenclature reflects its anatomical location (medial to the calcaneal apophysis) and developmental origin, following established conventions in anatomical terminology, such as os tibiale externum, which follows the standard pattern of naming by accessory bone, anatomical location, and specific detail [10, 11]. The biomechanical significance of the calcaneal apophysis has been well established in previous research [8, 12], with particular emphasis on their role as critical attachment sites for the abductor hallucis, flexor digitorum brevis, and plantar aponeurosis at the calcaneal apophysis because of their susceptibility to increased stress during periods of accelerated bone growth, which often outpaces muscle development, leading to heightened traction forces and muscle-tendon imbalances.

Comprehensive biomechanical studies have revealed significant implications of lower extremity alignment on heel loading patterns [4, 5, 9]. Research has demonstrated that a hindfoot eversion of 5 degrees generates a 15% increase in medial heel pressure [4], and the medial heel experiences 11-12% higher pressure compared to the lateral heel in patients with valgus alignment [4]. Furthermore, investigations have documented altered pressure distributions in patients with hindfoot varus deformity, showing significant lateral displacement of forefoot loading patterns compared to normal controls [5, 9]. These modified pressure patterns may significantly influence apophyseal development and fusion sequences [3], with the medial process being particularly susceptible due to its anatomical position and role in force transmission during gait.

The symmetrical presentation of unfused medial processes in this case represents a notable deviation from established developmental patterns. Previous studies have documented that under normal circumstances, the medial process initiates development at approximately age 9-10, with fusion occurring approximately 12-24 months after initial ossification [2, 13]. The typical timeline of ossification has been well established, with complete fusion observed between ages 14 and 16 in females [1, 2, 13]. The bilateral persistence of unfused apophysis in our patient deviates significantly from this expected timeline, suggesting potential developmental implications of chronic biomechanical alterations. In the assessment of pediatric foot ossification, MRI would indeed be superior to CT for evaluating cartilaginous structures [14]. However, an MRI was not performed due to the clinician's determination that its low cost-effectiveness for incidentally found normal variations would not provide additional information that would significantly impact treatment decision-making.

The clinical significance of this anatomical variation warrants careful consideration [10, 11]. While mechanical stress on the calcaneal apophysis has been well-documented in conditions, such as Sever's disease [1, 15], the developmental consequences of chronic biomechanical alterations from lower extremity malalignment represent a distinct clinical entity [16]. The selective non-fusion pattern observed in this case, specifically localized to the medial process bilaterally, differs from previously documented pathological conditions of the calcaneal apophysis [1, 3].

This report documents a previously unrecognized pattern of bilateral unfused medial processes of the calcaneal apophysis in association with coxa valga and genu valgum. While the limitations inherent in a single case observation preclude definitive conclusions regarding causality, the distinctive features of this case, including the bilateral symmetry, anatomical location, and concurrent lower extremity malalignment, suggest potential developmental implications that merit further investigation through systematic studies.

## CONCLUSION

This radiologic case report documents a distinctive developmental variation of the calcaneal apophysis characterized by bilateral unfused medial processes in a patient with idiopathic coxa valga and genu valgum. The anatomical features of these ossicles, including their symmetric morphology, precise location, and well-defined margins, represent a previously unreported developmental pattern that expands our understanding of calcaneal apophyseal variations. The concurrent presence of lower extremity malalignment in this case raises questions regarding potential developmental influences on apophyseal fusion patterns. Awareness of this anatomical variant is clinically important to prevent misdiagnosis as fractures or pathological conditions in radiographic evaluations. Understanding the relationship between mechanical axis deviation and apophyseal development may have implications for the evaluation and management of patients with complex lower extremity malalignment.

## AUTHORS’ CONTRIBUTIONS

The author confirms sole responsibility for the following: Study conception and design, data collection, analysis and interpretation of results and manuscript preparation.

## Data Availability

All the data and supportive information are provided within the article.

## LIST OF ABBREVIATIONS

CT: Computed Tomography
MRI: Magnetic Resonance Imaging
VR: Volume Rendering

## STANDARDS OF REPORTING

CARE guidelines were followed in this study.
